# White matter alterations in drug-naïve children with Tourette syndrome and obsessive-compulsive disorder

**DOI:** 10.3389/fneur.2022.960979

**Published:** 2022-10-03

**Authors:** Komal Bharti, Giulia Conte, Silvia Tommasin, Costanza Giannì, Antonio Suppa, Giovanni Mirabella, Francesco Cardona, Patrizia Pantano

**Affiliations:** ^1^Department of Human Neuroscience, Sapienza University of Rome, Rome, Italy; ^2^Istituto di Ricovero e Cura a Carattere Scientifico (IRCCS) Neuromed, Isernia, Italy; ^3^Department of Clinical and Experimental Sciences Section, Brescia University, Brescia, Italy

**Keywords:** Tourette syndrome, obsessive-compulsive disorder (OCD), white matter (WM) microstructural organization, drug-naïve, diffusion tensor imaging (DTI), fractional anisotropy (FA)

## Abstract

Tourette syndrome (TS) and early-onset obsessive-compulsive disorder (OCD) are frequently associated and conceptualized as distinct phenotypes of a common disease spectrum. However, the nature of their relationship is still largely unknown on a pathophysiological level. In this study, early structural white matter (WM) changes investigated through diffusion tensor imaging (DTI) were compared across four groups of drug-naïve children: TS-pure (*n* = 16), TS+OCD (*n* = 14), OCD (*n* = 10), and 11 age-matched controls. We analyzed five WM tracts of interest, i.e., cortico-spinal tract (CST), anterior thalamic radiations (ATR), inferior longitudinal fasciculus (ILF), corpus callosum (CC), and cingulum and evaluated correlations of DTI changes to symptom severity. Compared to controls, TS-pure and TS+OCD showed a comparable pattern of increased fractional anisotropy (FA) in CST, ATR, ILF and CC, with FA changes displaying negative correlation to tic severity. Conversely, in OCD, FA decreased in all WM tracts (except for the cingulum) compared to controls and negatively correlated to symptoms. We demonstrate different early WM microstructural alterations in children with TS-pure/TS+OCD as opposed to OCD. Our findings support the conceptualization of TS+OCD as a subtype of TS while suggesting that OCD is characterized by independent pathophysiological mechanisms affecting WM development.

## Introduction

The clinical hallmark of Tourette syndrome (TS) is the presence of multiple motor and phonic tics lasting at least 1 year (1). Although tics are the defining feature, TS is a multifaceted neurodevelopmental disorder in which several other neuropsychiatric conditions may occur (2). Obsessive-compulsive disorder (OCD) is present in up to 61.5% of individuals with TS (3) and is characterized by higher genetic heritability and disease severity as compared to the adult form (4, 5). Evidence is accumulating in support for the common genetic liability of TS and OCD (6, 7), which has raised new interest for their comorbidity (TS+OCD), in terms of both clinical course and treatment. However, although TS and OCD are both thought to reflect brain network disorders (8–10), their clinical association and overlap have been poorly understood on a pathophysiological level, possibly because of the lack of studies comparing the comorbid condition with the “pure” phenotypes.

To fill this knowledge gap, we have recently used diffusion tensor imaging (DTI) and resting-state functional MRI (rsfMRI) to investigate structural as well as functional brain changes in drug-naïve children with TS, TS+OCD, and OCD (11, 12). Those studies demonstrated that TS+OCD shares common neural correlates with TS, but not with OCD, in terms of structural WM changes and functional connectivity abnormalities in fronto-cerebellar and fronto-parietal networks (11, 12). However, our previous DTI analysis (12) focused on cerebellar connections by studying WM microstructural integrity of the three cerebellar peduncles, thus not clarifying the possible pathophysiological contribution of early changes in supratentorial WM bundles in TS and OCD.

In the present study, we investigated microstructural changes in those brain WM bundles that previous studies found to be altered in TS and/or OCD (13–23). We studied pediatric drug-naïve TS patients without comorbidities (TS-pure), TS patients with OCD (TS+OCD) and pure OCD patients in comparison to a control group, to provide evidence on primary WM abnormalities and their relationship to clinical severity. Based on our prior works on the same patient cohort (11, 12), we hypothesize that: (i) TS-pure and TS+OCD share common microstructural WM alterations and may be regarded as a unitary group, and (ii) TS-pure/TS+OCD and OCD are underpinned by different WM changes. We also hypothesize that the degree of WM structural integrity in patients with TS and OCD may be predicted by severity measures of tics and obsessive-compulsive symptoms. The study has been designed to control for several confounding factors such as disease duration, comorbidities, medication, and age. In particular, pediatric drug-naïve populations with definite comorbidity profiles are crucial to identify primary neuroimaging correlates in neurodevelopment disorders such as TS and OCD.

## Methods

### Participants

Seventy drug-naïve children were recruited from a specialized outpatient clinic for TS and related disorders (Child and Adolescent Neuropsychiatry Unit, Department of Human Neurosciences, Sapienza University of Rome, Italy). Patients were diagnosed according to DSM-5 criteria by a child neuropsychiatrist experienced in TS, OCD, and related disorders. Nineteen participants received a diagnosis of TS-pure, 19 of TS+OCD, 17 of OCD. As controls, we selected 15 age-matched participants with episodic tension headache who were headache-free during the MRI scans. Symptom severity was assessed using the Yale Global Tic Severity Scale [YGTSS, total tic severity scale: max. 50, without impairment score, (24)] and the Children's Yale-Brown Obsessive-Compulsive Scale [CY-BOCS, (25)]. Children diagnosed with TS and CY-BOCS scores above 14 were identified as patients with the comorbid condition and included in the TS+OCD group. All subjects had normal cognitive profile (IQ ≥ 70) according to Wechsler Intelligence Scale for Children III (WISC-III) full scale and were right-handed, as assessed by the Edinburgh handedness inventory (26). Other developmental disorders (e.g., Attention-Deficit/Hyperactivity Disorder, ADHD) or psychiatric disorders were excluded using the Schedule for Affective Disorders and Schizophrenia [K-SADS-PL, (27)] administered to both parents. Parents or guardians provided written informed consent. The study was approved by the Institutional Review Board and conformed to the Declaration of Helsinki.

### MRI parameters

After clinical evaluation, all participants underwent an MRI scan performed with 3.0 T scanner (Magnetic Verio; Siemens, Erlangen, Germany) with a 12-channel head coil designed for parallel imaging (GRAPPA, Generalized Autocalibrating Partial Parallel Acquisition) using a standardized protocol. Noise reduction headphones were used to prune the scanner noise. Head positioning was standardized using canthomeatal landmarks, and the head motion was minimized by inserting foam pads between the participant's head and the head coil. Moreover, participants were instructed to lie as still as possible during and between scans. MRI included: (i) Diffusion tensor imaging (DTI, single-shot echo-planar spin-echo sequence with 30 directions, TR = 12.200 ms, TE = 94 ms, FOV = 192 mm^2^, matrix = 96 × 96, b = 0 and 1,000 s/mm^2^, axial 2-mm-thick slices, no gap); (ii) 3D T1-weighted MPRAGE (TR = 1,900 ms, TE = 2.93 ms, 176 sagittal 1-mm-thick sections, without a gap, flip angle = 9°, FOV = 260 mm^2^, matrix = 256 × 256); (iii) dual turbo spin-echo proton density and T2- weighted images (DP-T2) (TR = 3,320 ms, TE = 10/103 ms, FOV = 220 mm^2^, matrix = 384 x 384, 25 axial 4-mm thick slices, 30% gap) acquired to rule out possible concomitant brain focal lesions.

### Data analysis

#### Preprocessing

MRI preprocessing was performed *via* FMRIB's software library (FSL), version 5.0.9 (http://fsl.fmrib.ox.ac.uk). Considering that the prevalence of motion in the child population is high in neuroimaging data (28), the DTI scan of each subject was first visually inspected and entirely removed in presence of artifacts even in one single volume alone. Secondly, using the FSL tools “eddy” and “topup,” correction of the susceptibility-by-movement interactions was performed. Eddy current corrected DTI files were further subjected to DTI Fit using FMRIB's diffusion toolbox to generate fractional anisotropy (FA), mean diffusivity (MD), axial diffusivity (AD), and radial diffusivity (RD) maps.

#### Tract–based spatial statistics

Firstly, FA maps were subjected to the Tract-Based Spatial Statistical (TBSS) tool for the voxel-wise statistical analysis (28). To deal with age-dependent skull characteristics, we created a pediatric skeleton template by averaging the FA maps, thresholded at mean FA = 0.2 and binarized, of all participants in the same space of a reference subject. Mean FA derived from the FA of the patients was therefore used as a pediatric template. In order to run the TBSS, linear registration was automatically performed to register FA images of all participants onto the pediatric template. Similarly, MD, AD, and RD maps were registered to the pediatric template to obtain comparable images in the same standardized space.

We selected five WM tracts chosen to be of interest (TOIs) (29) according to previous findings showing consistent WM bundles alterations in TS or OCD patients (14, 17, 20, 22, 30, 31). The five TOIs, i.e., anterior thalamic radiations (ATR), corpus callosum (CC), cortico-spinal tract (CST), inferior longitudinal fasciculus (ILF), and cingulum, were first selected in MNI standard space using the JHU ICBM-DTI-81 White-Matter atlas and then were linearly registered onto the pediatric template space. The TOIs in the pediatric template space were further used to create binarized masks for further voxel-wise inter-group analysis.

#### Statistical analysis

We performed the Shapiro-Wilk normality test to check for normal distribution of the demographic and clinical data. One-way ANOVA and *post-hoc* unpaired *t-*tests (two-tailed, alpha = 0.05, unequal variance) were performed to investigate differences among groups with respect to age. The Chi-square test was also exploited to check for sex distribution among the groups. Differences in clinical scores among pure TS, TS+OCD, and OCD were analyzed with the Mann-Whitney U test. Analyses were performed with SPSS (Statistical Package for the Social Sciences, https://www.ibm.com/analytics/spss-statistics-software).

Using the FSL Randomize (*n* = 5,000 permutations), non-parametric statistics were performed to investigate voxel-wise differences within the five pre-selected microstructural WM tracts between pairs of the study cohorts and to further compute correlations with clinical measures (i.e., YGTSS and CYBOCS). Statistical significance was set at *p* < 0.05, corrected for false discovery rate (FDR). Age and sex were included as covariates of no interest.

## Results

### Demographic and clinical characteristics

The clinical details of the study cohorts are summarized in Table 1. From the original population of 70 children, 51 were included in the study. Overall, 19 children were excluded due to excessive head motion (*n* = 10) or inability to complete the MRI scan (*n* = 9). Children who excessively moved their head were mainly diagnosed with TS or TS+OCD (respectively 3 and 4 cases), while two children were diagnosed with OCD, and one was an healthy control. On the contrary, 5 children with OCD, one child with TS+OCD and 3 controls could not complete the scan. Sixteen subjects were included in the TS-pure group (15 males, mean age ± standard deviation: 9.7 ± 2.1 years), 14 in TS+OCD (10 males, 10.2 ± 2.1 years old), 10 in OCD (7 males, 10.9±2.5 years old) and 11 age-matched children in the control group (2 males, 9.9 ± 1.2 years old).

**Table 1:** Demographic and clinical characteristics.

**Variables**	**TS-pure** ***N*** **= 16** **Mean ±SD**	**TS+OCD** ***N*** **= 14** **Mean ±SD**	**OCD** ***N*** **= 10** **Mean ±SD**	**Controls** ***N*****= 11** **Mean ±SD**	**TS-pure vs. controls**	**TS+OCD *vs*. controls**	**OCD vs. controls**	**TS-pure vs. OCD**	**TS+OCD vs. OCD**	**TS-pure vs. TS+OCD**
Age	9.7 ± 2.1	10.2 ± 2.1	10.9 ± 2.5	9.9 ± 1.3	*p* = 0.30	*p* = 0.43	*p* = 0.31	*p* = 0.18	*p* = 0.46	*p* = 0.44
Gender (male/female)	15/1	10/4	7/3	2/9	*p* < 0.001^*^	*p* = 0.008^*^	*p* < 0.001^*^	*p* = 0.10	*p* = 0.94	*p* = 0.10
YGTSS score (0–50)	17.5 ± 6.7	18.1 ± 10.8	0.8 ± 1.7	–	–	–	–	*p* < 0.001^*^	*p* < 0.001^*^	*p* = 0.88
CYBOCS score (0–40)	0.25 ± 0.7	16.4 ± 6.1	18.6 ± 7.5	–	–	–	–	*p* < 0.001^*^	*p* = 0.53	*p* < 0.001^*^

TS-pure, children with pure Tourette syndrome; TS+OCD, children with Tourette syndrome and obsessive-compulsive disorder; OCD, children with pure obsessive-compulsive disorder; Controls, control group; YGTSS, Yale Global Tic Severity Scale; CYBOCS, Children's Yale-Brown Obsessive-Compulsive Scale.

Values are reported as mean ± SD.

Differences in the demographic and clinical scores were assessed by Mann Whitney (U) test.

Differences in the gender were assessed by chi square (χ2) test.

* Significant p-values (p < 0.05).

TS-pure, TS+OCD, OCD and controls were not statistically different for age [F (3, 46) = 0.77, *p* = 0.51, partial η2 = 0.048). Conversely, chi-square test revealed uneven sex distribution between TS-pure and controls [χ2 (1, *N* = 27) = 15.90, *p* < 0.001], and between OCD and controls [χ2 (1, *N* = 21) = 48.90, *p* < 0.001]. Mann-Whitney U test revealed no significant difference in YGTSS scores between TS-pure and TS+OCD (U = 108.5, *p* = 0.88), as well as in CY-BOCS scores between OCD and TS+OCD (U = 65, *p* = 0.53).

### WM microstructural alterations in TS, TS+OCD and OCD

**Figure 1:**
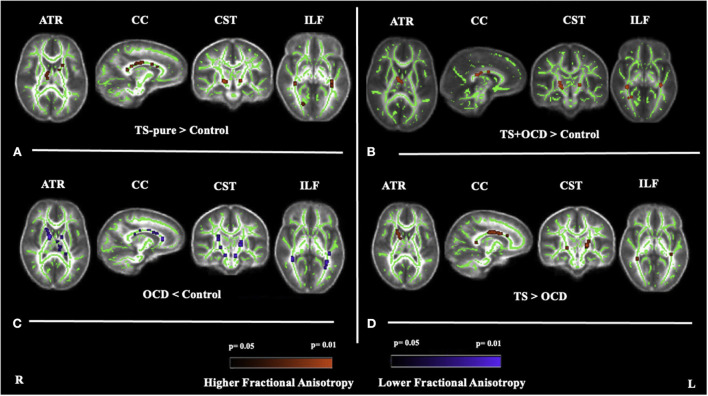
Fractional anisotropy (FA) differences between **(A)** TS-pure and controls, **(B**) TS+OCD and controls, **(C)** OCD and controls, **(D)** TS and OCD, at anterior thalamic radiation (ATR), corpus callosum (CC), corticospinal tract (CST), inferior longitudinal fasciculus (ILF). **(A)**: higher FA in TS-pure than in controls, **(B)**: higher FA in TS+OCD than in controls, **(C)**: lower FA in OCD than in controls, **(D)**: higher FA in TS than in OCD. Results were obtained within the mask of ATR, CC, CST, and ILF. Results are presented in the whole brain FA skeleton mask derived from the complete set of participants. FA results were corrected for multiple comparisons at the false discovery rate (FDR) of *p* < 0.05. Red color: Higher FA differences, Blue color: Lower FA differences, TS-pure: participants with pure Tourette syndrome (TS), OCD: participants with obsessive compulsive disorder, TS+OCD: TS participants with comorbid condition, TS: participants with TS-pure and TS+OCD.

TS-pure patients showed higher FA and lower MD, AD, RD than controls within the right ATR, the genu, body, splenium of CC, the CST, and the ILF bilaterally (Figure 1A). Since the voxel-wise results obtained by analyzing MD, AD, and RD perfectly matched those obtained by FA analysis, the relative maps are shown as Supplementary material.

TS+OCD patients exhibited overlapping DTI features, e.g., FA, MD, AD, RD, as TS-pure in respect to controls (Figure 1B), i.e., DTI parameters were comparable in TS-pure and TS+OCD within any of the investigated WM tracts. Based on such findings, TS-pure and TS+OCD were merged for the following analyses into a joint group named “TS.”

OCD patients showed opposite DTI alterations with respect to controls, i.e., lower FA and higher MD, AD, RD within the ATR bilaterally, the genu, body, splenium of CC, the CST and ILF bilaterally (Figure 1C).

The direct comparison between TS and OCD revealed that TS had higher FA and lower MD, AD, RD than OCD within the four aforementioned WM bundles (Figure 1D).

No changes in DTI parameters were found in the cingulum in any group comparison.

White matter regions exhibiting differences in FA between groups are detailed in Table 2. MD, AD, RD alterations in all the four group comparisons are available as Supplementary Figures 1–3.

**Table 2:** White matter tracts with fractional anisotropic (FA) differences in TS and OCD.

**Group differences**	**Brain areas within the tract**	**No of clusters**	**Clusters voxels**	**Coordinates**			Peak–t stat^^*^^
				**X**	**Y**	**Z**	
**TS-pure>controls**							
ATR	Right anterior limb of internal capsule Left anterior limb of internal capsule	20 10	10 7	−19.9 −5.97	28.1 −33.4	−25.7 −65.4	4.15 3.95
CC	Splenium Body Genu	20 14 5	10 10 10	−9.84 9.32 12.1	−37.5 −10.9 25.1	−19.7 −13.7 −19.7	3.10 2.93 2.45
CST	Right cerebral peduncle Left cerebral peduncle	6 5	10 10	11.5 −11.7	−18.6 −18.6	−52.5 −53.4	2.74 2.61
ILF	Right inferior fronto-occipital fasciculus Left inferior fronto-occipital fasciculus Right posterior thalamic radiation	12 14 7	8 10 8	34.6 −31.7 28.9	−42.4 −42.2 −67.6	−39.7 −39.7 −37.3	3.34 3.26 2.91
**TS±OCD>Controls**							
ATR	Right anterior limb of internal capsule	20	15	11.2	−6.87	−31.7	4.16
CC	Splenium Body	19 15	10 7	−7.65 6.55	−43.4 −6.81	−27.7 −15.5	3.11 3.53
CST	Right cerebral peduncle Left cerebral peduncle Right Corticospinal tract Left Corticospinal tract	14 13 20 7	10 5 25 10	17.6 −17.4 14.3 −13.6	−17.2 −17.2 −16.7 −16.7	−39.2 −10.3 −48.7 −44.0	3.28 3.20 2.91 3.15
ILF	Right inferior fronto-occipital fasciculus Left inferior fronto-occipital fasciculus	13 10	10 8	35 −33.8	−33.9 −31.5	−44 −44	3.32 2.94
**OCD < Controls**							
ATR	Right anterior limb of internal capsule Left anterior limb of internal capsule	20 7	25 10	13.2 −13.4	4.62 −5.8	−28.8 −28.8	4.34 2.70
CC	Splenium Body Genu	10 5 25	15 12 35	−13.4 −13.4 −13.4	−55.2 −24.3 23.1	−22.6 11.2 −27.8	3.88 4.15 3.69
CST	Right corticospinal tract Left corticospinal tract Right cerebral peduncle Left cerebral peduncle	30 30 15 12	25 20 12 10	24.3 −23.1 4.87 −8.41	−26.7 −26.7 −26.7 −26.7	−21.2 −27.8 −70.1 −62.0	2.37 3.19 3.64 2.50
ILF	Right inferior fronto-occipital fasciculus Left inferior fronto-occipital fasciculus Right posterior thalamic radiation Left posterior thalamic radiation	10 15 20 30	11 10 40 35	33.89 −41.5 27.4 −24.3	−40.5 −21.0 −69.1 −64.3	−38.3 −44.5 −45.4 −45.4	2.47 2.32 3.47 2.69
**TS>OCD**							
ATR	Right anterior limb of internal capsule	21	15	12.26	−0.13	−28.3	2.48
CC	Splenium Body	7 17	5 15	−2.96 −2.01	−41 −3.96	−26.4 −18.8	3.26 4.10
CST	Right corticospinal tract Left corticospinal tract	5 15	2 10	12.9 −16	−21.0 −17.7	−50.6 −37.3	3.42 2.98
	Right cerebral peduncle Left cerebral peduncle	10 5	8 5	5.82 −7.94	−25.3 −22	−60.1 −62	2.94 3.60
ILF	Right inferior fronto-occipital fasciculus	5	7	35	−40.1	−41.1	3.18
	Left inferior fronto-occipital fasciculus	5	10	−34.9	−34.8	−41.1	2.96

After having registered the pediatric template, that we used for the second level analysis, on MNI space, coordinates were extracted from MNI 152 space.

TS-pure>controls represents higher fractional anisotropy (FA) in patients with pure Tourette than in controls. TS+OCD>controls represents higher FA in Tourette patients with comorbid obsessive-compulsive disorder than in controls. OCD < controls represents lower FA in patients with obsessive-compulsive disorder than in controls. TS>OCD represents higher FA in Tourette patients (TS-pure and TS+OCD) than in patients with obsessive-compulsive disorder. ATR, Anterior thalamic radiation; CC, Corpus Callosum; CST, Corticospinal tract; ILF, Inferior longitudinal fasciculus.

Results were corrected for false discovery rate (FDR) at p < 0.05.

* Peak t-stat denotes the maximum statistical value (t-stat) for the peak activity.

### Clinical-neuroradiological correlations

**Figure 2:**
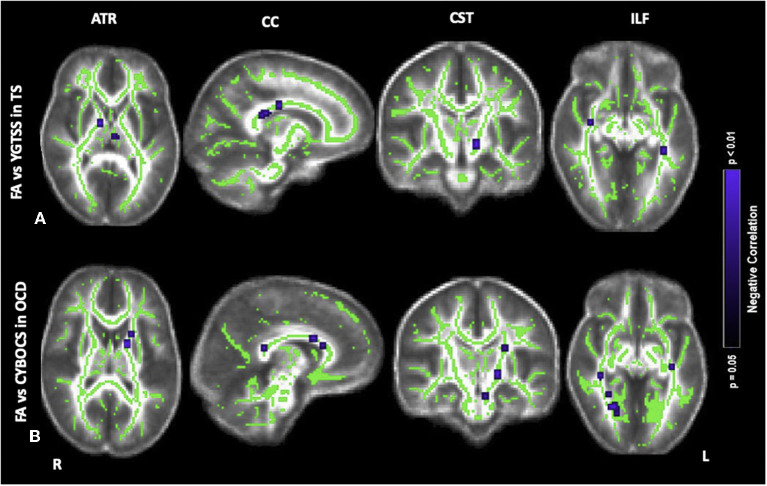
Correlations of fractional anisotropy (FA) abnormalities in **(A)** TS with YGTSS, **(B)** OCD with CYBOCS at the anterior thalamic radiation (ATR), corpus callosum (CC), corticospinal tract (CST), inferior longitudinal fasciculus (ILF). Results were obtained within the mask of ATR, CC, CST, and ILF. Results are presented in the whole brain FA skeleton mask derived from the complete set of participants. Results were corrected for multiple comparisons at the false discovery rate (FDR) of *p* < 0.05. Blue color: Negative correlation, TS: participants with pure Tourette and comorbid condition [TS+(TS+OCD)], OCD: participants with obsessive compulsive disorder, YGTSS: Yale Global Tic Severity Scale, CYBOCS: Children's Yale Brown Obsessive-Compulsive Scale.

In Figures 2A,B, the correlations between FA alterations with clinical scores are displayed for TS and OCD groups. For both TS and OCD, the maps of MD, AD, RD alterations and correlation with clinical severity are shown as Supplementary Figure 4.

Concerning the severity of tics scored by the YGTSS, in the TS group, there was a negative correlation between FA and YGTSS within ATR (i.e., anterior limb of internal capsule), CC (i.e., body, splenium, genu), CST (i.e., cerebral peduncles) and ILF (i.e., posterior thalamic radiation, and inferior fronto-occipital fasciculus) (Figure 2A). Accordingly, within the same tracts, a positive correlation was found between the other diffusivity parameters, i.e., MD, AD, RD, and YGTSS. No significant correlation was observed between DTI parameters and YGTSS in any of the five selected WM tracts when TS-pure and TS+OCD were analyzed separately.

Concerning the severity of OCD symptoms as tested by the CY-BOCS, in the TS+OCD, there was no significant correlation between DTI parameters and CY-BOCS scores. In OCD, CY-BOCS negatively correlated with FA and positively with MD, AD, and RD within ATR (i.e., anterior limb of internal capsule), CC (i.e., body, splenium, genu), CST (i.e., cerebral peduncles), and ILF (i.e., posterior thalamic radiation, and inferior fronto-occipital fasciculus) (Figure 2B).

Correlational analyses of FA with clinical scales are reported in Table 3.

**Table 3:** Brain areas within white matter tracts showing significant correlations between fractional anisotropy (FA) and clinical data.

**Clinical parameters**	**Brain areas within the tract**	**No of clusters**	**Clusters voxels**	**Coordinates**			Peak–t stat^^*^^
				**X**	**Y**	**Z**	
**FA vs. YGTSS in Tourette (TS)**							
ATR	Right anterior limb of internal capsule	26	10	8.45	−6.31	−38.3	1.20
CC	Splenium Body	26 12	12 10	3.22 3.22	−29.1 −13.4	−23.1 17.4	1.15 1.30
CST	Left cerebral peduncle	20	10	−8.89	−26.2	−56.8	1.07
ILF	Right posterior thalamic radiation Left inferior fronto-occipital fasciculus	11 13	9 15	30.8 −36.7	−10.1 −37.2	−46.8 −46.8	1.16 1.05
**FA vs. CYBOCS in obsessive compulsive disorder (OCD)**							
ATR	Left anterior limb of internal capsule	15	10	−15.3	−3.9	−28.3	1.20
CC	Splenium Body Genu	5 10 5	10 5 10	−8.19 −8.19 −8.19	−41.9 −2.5 16.4	−25.9 −15.0 −25.5	1.23 1.05 1.69
CST	Left Corticospinal Tract Left cerebral peduncle	13 15 10	9 10 7	−23.1 −16.1 −7.94	−19.1 −19.1 −19.1	−15.5 −42.5 −59.6	1.25 1.89 1.30
ILF	Left inferior fronto-occipital fasciculus	17 13 25	15 10 40	36.4 31.7 27.9	−30.1 −46.2 −68.1	−38.8 −38.8 −38.8	1.99 1.56 1.85
	Right posterior thalamic radiation	10	9	−33.3	−44.8	−38.8	1.23

After having registered the pediatric template, that we used for the second level analysis, on MNI space, coordinates were extracted from MNI 152 space.

FA vs. YGTSS in Tourette (TS) represents negative correlations between FA values and YGTSS in TS patients at ATR, CC, CST, ILF.

FA vs. CYBOCS in Obsessive compulsive disorder (OCD) represents negative correlations between FA values and CYBOCS in OCD patients at ATR, CC, CST, ILF.

FA, fractional anisotropy; YGTSS, Yale Global Tic Severity Scale; CYBOCS, Children's Yale Brown Obsessive-Compulsive Scale; ATR, Anterior thalamic radiation; CC, Corpus Callosum; CST, Corticospinal tract; ILF, Inferior longitudinal fasciculus.

Results were corrected for false discovery rate (FDR) at *p* < 0.05.

* Peak-t stat denotes the maximum statistical value (t-stat) for the peak activity.

## Discussion

The present DTI study extends our prior observations (11, 12) demonstrating abnormalities in several WM tracts in drug-naïve TS-pure, TS+OCD, and OCD. Such WM microstructural changes represent early-stage correlates, which are not affected by long disease duration, medication, or any other comorbidity.

### WM microstructural alterations in TS

TS-pure and TS+OCD showed common DTI changes with respect to controls, i.e., increased FA in the corpus callosum, anterior thalamic radiations, corticospinal tract, and inferior longitudinal fasciculus. Previous studies in children with TS reported variable DTI findings, i.e., increased FA (32), reduced FA (15, 33), or no variation at all (22, 34). Despite such inconsistencies, evidence from prior works in children are in agreement with our results converging on early structural abnormalities in three major regions, i.e., interhemispheric bundles (13, 15, 20, 22, 32, 33), main motor pathways (13, 17, 20, 32) and prefrontal and fronto-striatal pathways (18, 35). In adult TS studies, results on DTI changes are also heterogeneous, showing either an increased (36, 37) and a decreased FA (38, 39) of the interhemispheric bundles and WM tracts of sensorimotor regions. The factor age is critical to explain such inconsistencies. The WM undergoes age-dependent changes throughout the lifespan (40), making comparisons between pediatric and adult cohorts hardly affordable. Moreover, given that less than 25% of individuals with TS encounter a significant persistence of tics into adulthood (41), adults may be conceived as a peculiar subpopulation with different or more pronounced neural abnormalities. Comorbidities–which were poorly controlled in former pediatric investigations–represent another relevant aspect contributing to heterogeneous findings. To control for this aspect, we have carefully selected participants based on comorbid conditions and included only treatment-naïve children. To our knowledge, the only previous pediatric study with a similar design is that of Wolff et al. (22), which did not show FA difference in the CC of boys with pure TS compared to controls. Differences in DTI analyses procedure, in particular the selection of a definite TOI, which was further partitioned into five segments (or sub-TOIs), might explain the discrepancy with our results.

The increased FA in the CC of children with TS, that we found, may be indicative of stronger structural connectivity due to increased axonal density, thicker axons, greater myelination, or a combination of these processes. In normal neurodevelopment, there is a linear relationship between interhemispheric connectivity and motor learning/control (42–44). The observed increased connectivity in callosal fibers, which was negatively correlated to tic severity, raises the intriguing possibility of enhanced interhemispheric communication between areas involved in motor control and tic inhibition in children with TS. Moreover, the presence of atypical WM structure in the ATR, which is the major fiber bundle connecting the prefrontal cortex with the thalamus through the anterior limb of the internal capsule, greatly supports the cortico-striatal-thalamic circuitry model of TS, which is considered the leading pathophysiological account of the disorder (45, 46). Lastly, increased FA was also identified in the ILF of TS children. ILF is a large associative bundle connecting the occipital with the temporal lobe, and it is critically involved in visually-guided behaviors and object recognition (47–50). Our finding of increased FA in ILF might explain the enhanced abilities in visuomotor integration and learning in patients with TS, which have been described on both behavioral (51, 52) and neurophysiological levels (53).

Overall, the observation that lower FA was associated with greater tic severity, strongly suggest an inverse relationship between WM organization in TS and disorder expression. Thus, by detecting common structural correlates, in line with our previous findings (11, 12), our data support the conceptualization of TS+OCD as a specific subtype of TS, in which obsessive-compulsive symptoms may be conceived as heterotypic manifestations of a spectrum rather than the expression of a distinct disorder.

### WM microstructural alterations in OCD

Children with pure OCD manifested opposite FA changes compared to the TS group, suggesting different WM changes sustain two disorders. To date, several DTI meta-analyses have been conducted in OCD, albeit they have many inconsistencies. In a meta-analysis combining data from pediatric and adult cohorts (54), the most prominent and replicable result was a decreased FA in the genu of CC and left orbito-frontal WM. Conversely, another metanalytic investigation analyzing pediatric and adult studies separately (55) showed no FA alterations in children but decreased FA in the genu and anterior body of CC in adults. In sum, this latter study suggested the existence of different pathophysiological processes in early onset compared to adult OCD. Similarly, no FA changes were detected in a large cohort of children and adolescents in a study from the ENIGMA OCD working group (31). However, a previous study by Fitzgerald et al. (56) specifically addressed the effects of age on FA in children and adolescents with OCD. The authors showed an FA reduction of the CC in children aged 8–11 years and an FA increase in adolescents aged 16–19 years. Thus, WM organization likely undergoes different developmental phases in OCD, which are sustained by differing patterns of pruning and myelination according to age. Therefore, inconsistencies across DTI findings, as well as the null results from metanalyses, might be explained according to neurodevelopmental features, especially when pediatric broad-aged cohorts are considered.

In our study, FA changes in the anterior CC and ATR agrees with previously reported evidence of abnormal function and structure in the cortico-striatal-thalamic circuitry in OCD (57–59). The anterior CC contains WM fibers projecting to the prefrontal regions and connecting the right and left prefrontal, premotor and supplementary motor cortex, all areas repeatedly shown to have aberrant function and volume in OCD [e.g., (60)]. The ATR contains WM pathways connecting the frontal lobe and thalamus. Thus, anterior CC and ATR collectively represent key traits of cortico-striatal-thalamic circuitry, on which pathophysiological models of OCD are built. Compared to controls, OCD children also exhibited decreased FA within the ILF. Such findings indirectly suggest abnormalities in WM areas relevant for visuo-spatial abilities, which have been repeatedly reported to be impaired in OCD patients (61) and have been associated with the persistence of OCD in adult age (62).

Overall, the observed negative correlation of obsessive-compulsive symptoms with FA values in the CST, ATR, CC, and ILF indicate that more severe clinical phenotypes are underpinned by less organized WM tracts in OCD children.

From the original OCD group, a few children were affected by head motion during MRI acquisition leading to question whether FA reductions were due to motion artifacts (63). However, visual inspection procedure was performed to identify artifacts on all subjects, irrespectively of group membership (TS, OCD, or controls). Furthermore, we might expect that, unlike TS, the scans of OCD patients were less likely corrupted by motion effects as a consequence of the clinical symptoms and showed the same probability of motion artifacts as the pediatric healthy population with similar age. As a matter of fact, TS patients showed FA increments in respect to controls, therefore FA reduction in patients with OCD appears to be inherently associated with the disorder, rather than to head motion.

### Differences in WM microstructural alterations in TS and OCD

When looking at the specific difference between clinical groups, a dichotomic pattern of WM abnormalities within ATR, CST, and ILF emerged in TS vs. OCD, i.e., increased FA in TS as opposed to decreased FA in OCD. This further supports the hypothesis that pure OCD represents a different entity from OCD in the context of TS, reflecting independent neuroadaptive processes. Interestingly, the TS group showed a negative correlation between FA and YGTSS, pointing to the idea that an early increase in axons, fiber density bundles and/or myelination in TS may be indicative of a compensatory reorganization in response to the disease pathophysiology. In the OCD group, the clinical-neuroradiological correlations suggest opposite considerations, as reduced fiber myelination and organization are associated with the overall disease burden.

A final comment concerns the DTI indexes other than FA such as MD, AD, and RD, collected in the TS and OCD cohorts. It is known that FA reflects myelination and organization of axon fibers (64, 65). Decreased FA would point to less myelinated and less compact WM tracts and vice versa (66, 67). However, given that the interpretation of FA changes is not univocal (68), additional DTI parameters such as MD, RD, and AD might help characterize WM microstructural abnormalities. RD and AD provide measures of myelin and axonal integrity, respectively (69, 70). In the present study, MD, RD, and AD scaled in the same direction within each clinical cohort, i.e., they all resulted decreased in TS and increased in OCD. Overall, these results further support our hypothesis of a differential organization and maturation of WM fibers rather than selective damage to axons or myelin in both TS and OCD.

Some limitations of the current study should be stressed. First, differences in sex distribution between patients and controls may have influenced the results. However, the effects of sex on our DTI findings have been minimized by including such variable as a nuisance covariate in the analysis. Thus, we believe that sex distribution has unlikely influenced our results. Second, the relatively small number of participants would preclude the immediate generalization of our findings to all children with TS and OCD. However, given the specific participants' age range and their careful selection, we believe that our results truly capture the WM microstructural organization in the early stages of TS and OCD, uninfluenced by other comorbidities, long disease duration and pharmacological treatment.

## Conclusion

This is the first study to characterize and compare WM microstructure in drug-naïve TS and OCD children. We highlight a shared pattern of WM microstructural changes in pure TS and combined TS+OCD as opposed to pure OCD, pointing to the conceptualization of TS+OCD as a peculiar subtype of TS. Compared to the normative population, the overall TS group showed a unique pattern of increased FA in callosal WM and in tracts linking the frontal, occipital and temporal cortices with each other and with the thalamus. The increased WM connectivity–which inversely correlated to tic severity–may represent an adaptive reorganization to aberrant or overactive sensory-motor processing in TS, possibly allowing partial compensation of tics. Conversely, children with OCD showed widespread reduced WM connectivity of callosal, temporo-occipital, and fronto-thalamic bundles, which were all related to greater disease severity and appear to play a role in the disease pathophysiology since an early stage.

## Data availability statement

The raw data supporting the conclusions of this article will be made available by the authors, without undue reservation.

## Ethics statement

The studies involving human participants were reviewed and approved by Ethics Committee of Azienda Ospedaliero-Universitaria Policlinico Umberto I. Written informed consent to participate in this study was provided by the participants' legal guardian/next of kin.

## Author contributions

KB: investigation, writing–original draft, writing–review and editing, MRI acquisition, data analysis, and data curation. GC: investigation, writing–original draft, writing–review and editing, and data curation. ST and CG: writing–review and editing, MRI acquisition, and data analysis. AS and GM: investigation and writing–review and editing. FC: investigation, writing–review and editing, data curation, conceptualization, design of the study, and study supervision. PP: investigation, writing–review and editing, MRI acquisition, data analysis, conceptualization, design of the study, and study supervision. All authors contributed to the article and approved the submitted version.

## Conflict of interest

The authors declare that the research was conducted in the absence of any commercial or financial relationships that could be construed as a potential conflict of interest. The reviewer LM declared a past collaboration with one of the authors AS to the handling editor.

## Publisher's note

All claims expressed in this article are solely those of the authors and do not necessarily represent those of their affiliated organizations, or those of the publisher, the editors and the reviewers. Any product that may be evaluated in this article, or claim that may be made by its manufacturer, is not guaranteed or endorsed by the publisher.

## Supplementary material

Supplementary Figure 1 Mean diffusivity (MD) differences between **(A)** TS-pure and controls, **(B)** TS+OCD and controls, **(C)** OCD and controls **(D)** TS and OCD at anterior thalamic radiation (ATR), corpus callosum (CC), corticospinal tract (CST), inferior longitudinal fasciculus (ILF). **(A)**: lower MD in TS-pure than in controls, **(B)**: lower MD in TS+OCD than in controls, **(C)**: higher MD in OCD than in controls, **(D)**: lower MD in TS than in OCD. Results were obtained within the mask of ATR, CC, CST, and ILF. Results were presented in the whole brain FA skeleton mask derived from the complete set of participants. MD results were corrected for multiple comparisons at the false discovery rate (FDR) of *p* < 0.05. Red: Higher MD differences, Blue: Lower MD differences, TS-pure: participants with pure Tourette syndrome (TS), OCD: participants with obsessive compulsive disorder, TS+OCD: TS participants with comorbid condition, TS: participants with TS-pure and TS+OCD.

Supplementary Figure 2 Axial diffusivity (AD) differences between **(A)** TS-pure and controls, **(B)** TS+OCD and controls, **(C)** OCD and controls **(D)** TS and OCD at anterior thalamic radiation (ATR), corpus callosum (CC), corticospinal tract (CST), inferior longitudinal fasciculus (ILF). **(A)**: lower AD in TS-pure than in controls, **(B)**: lower AD in TS+OCD than in controls, **(C)**: higher AD in OCD than in controls, **(D)**: lower AD in TS than in OCD. Results were obtained within the mask of ATR, CC, CST, and ILF. Results were presented in the whole brain FA skeleton mask derived from the complete set of participants. AD results were corrected for multiple comparisons at the false discovery rate (FDR) of *p* < 0.05. Red: Higher AD differences, Blue: Lower AD differences, TS-pure: participants with pure Tourette syndrome (TS), OCD: participants with obsessive compulsive disorder, TS+OCD: TS participants with comorbid condition, TS: participants with TS-pure and TS+OCD.

Supplementary Figure 3 Radial diffusivity (RD) differences between **(A)** TS-pure and controls, **(B)** TS+OCD and controls, **(C)** OCD and controls **(D)** TS and OCD at anterior thalamic radiation (ATR), corpus callosum (CC), corticospinal tract (CST), inferior longitudinal fasciculus (ILF). **(A)**: lower RD in TS-pure than in controls, **(B)**: lower RD in TS+OCD than in controls, **(C)**: higher RD in OCD than in controls, **(D)**: lower RD in TS than in OCD Results were obtained within the mask of ATR, CC, CST, and ILF. Results were presented in the whole brain FA skeleton mask derived from the complete set of participants. RD results were corrected for multiple comparisons at the false discovery rate (FDR) of *p* < 0.05. Red: Higher RD differences, Blue: Lower RD differences, TS-pure: participants with pure Tourette syndrome (TS), OCD: participants with obsessive compulsive disorder, TS+OCD: TS participants with comorbid condition, TS: participants with TS-pure and TS+OCD.

Supplementary Figure 4 Clinical correlations in TS and OCD with YGTSS and CYBOCS at the anterior thalamic radiation (ATR), corpus callosum (CC), corticospinal tract (CST), and inferior longitudinal fasciculus (ILF). **(A)**: positive correlation in TS between MD and YGTSS, **(B)**: positive correlation in OCD between MD and CYBOCS, **(C)**: positive correlation in TS between AD and YGTSS, **(D)**: positive correlation in OCD between AD and CYBOCS, **(E)**: positive correlation in TS between RD and YGTSS, **(F)**: positive correlation in OCD between RD and CYBOCS. Results were presented in the whole brain FA skeleton mask derived from the complete set of participants. Results were corrected for multiple comparisons at the false discovery rate (FDR) of *p* < 0.05. TS: participants with pure Tourette and comorbid condition [TS+(TS+OCD)]. OCD: participants with obsessive compulsive disorder, YGTSS: Yale Global Tic Severity Scale, CYBOCS: Children's Yale Brown Obsessive-Compulsive Scale.
