# Supplementary figures and images for: White matter alterations in drug-naïve children with Tourette syndrome and obsessive-compulsive disorder
Source: Front Neurol. 2022 Oct 3;13:960979. doi: 10.3389/fneur.2022.960979 (PMC9575657; doi:10.3389/fneur.2022.960979)

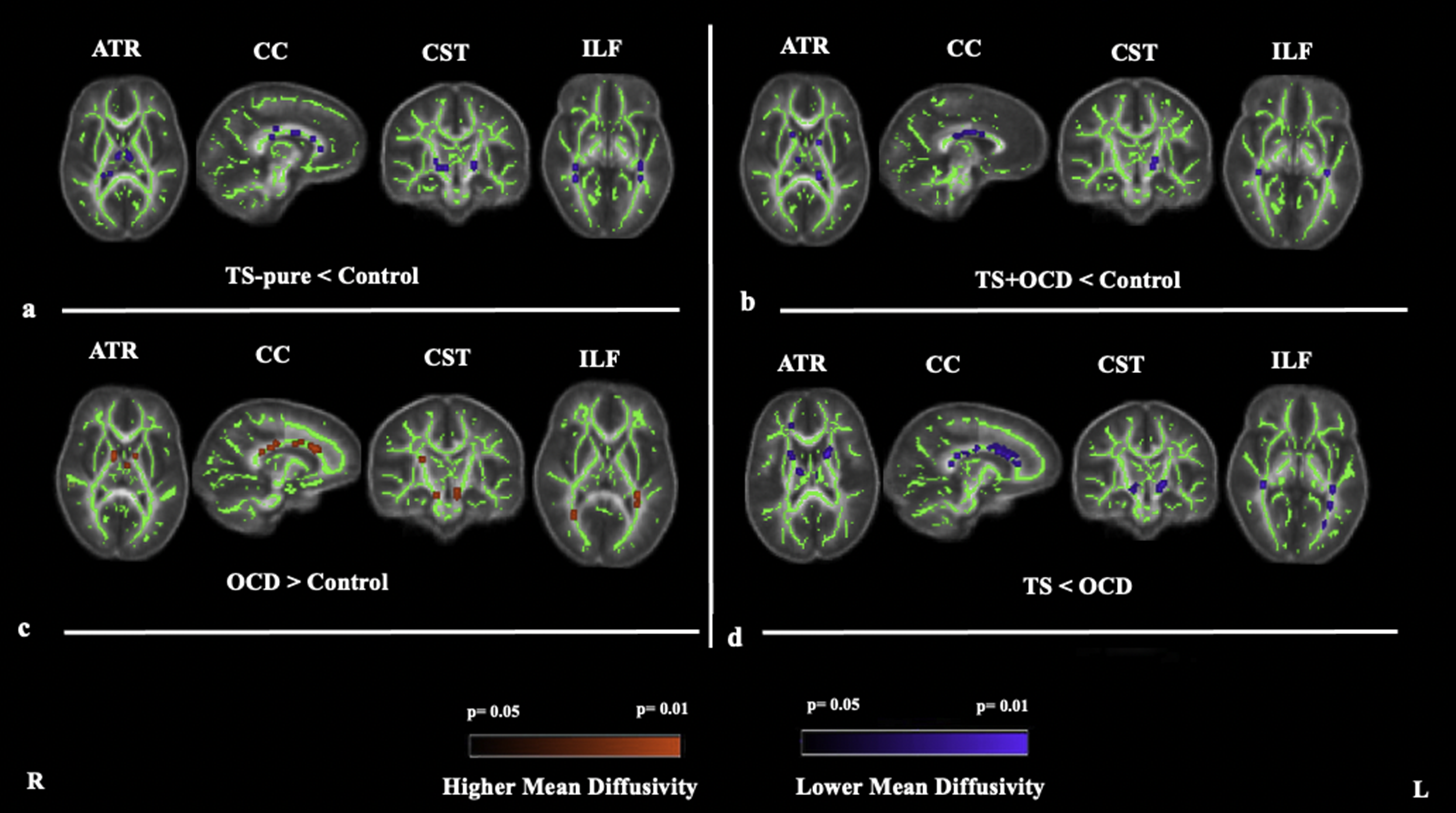

Supplement: Supplementary Figure 1 — Mean diffusivity (MD) differences between (A) TS-pure and controls, (B) TS+OCD and controls, (C) OCD and controls (D) TS and OCD at anterior thalamic radiation (ATR), corpus callosum (CC), corticospinal tract (CST), inferior longitudinal fasciculus (ILF). (A): lower MD in TS-pure than in controls, (B): lower MD in TS+OCD than in controls, (C): higher MD in OCD than in controls, (D): lower MD in TS than in OCD. Results were obtained within the mask of ATR, CC, CST, and ILF. Results were presented in the whole brain FA skeleton mask derived from the complete set of participants. MD results were corrected for multiple comparisons at the false discovery rate (FDR) of p < 0.05. Red: Higher MD differences, Blue: Lower MD differences, TS-pure: participants with pure Tourette syndrome (TS), OCD: participants with obsessive compulsive disorder, TS+OCD: TS participants with comorbid condition, TS: participants with TS-pure and TS+OCD. [file Image_1.TIFF]

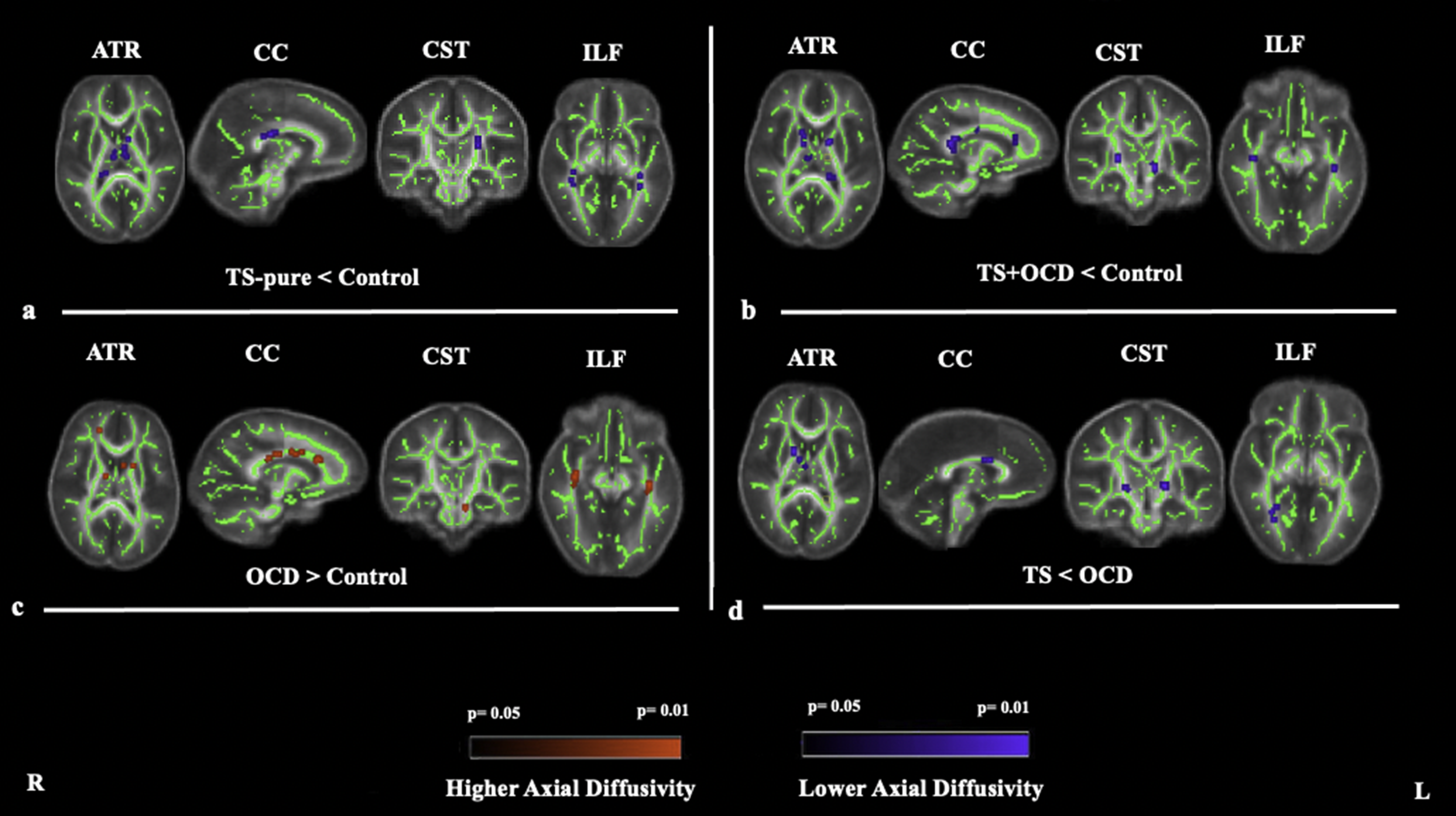

Supplement: Supplementary Figure 2 — Axial diffusivity (AD) differences between (A) TS-pure and controls, (B) TS+OCD and controls, (C) OCD and controls (D) TS and OCD at anterior thalamic radiation (ATR), corpus callosum (CC), corticospinal tract (CST), inferior longitudinal fasciculus (ILF). (A): lower AD in TS-pure than in controls, (B): lower AD in TS+OCD than in controls, (C): higher AD in OCD than in controls, (D): lower AD in TS than in OCD. Results were obtained within the mask of ATR, CC, CST, and ILF. Results were presented in the whole brain FA skeleton mask derived from the complete set of participants. AD results were corrected for multiple comparisons at the false discovery rate (FDR) of p < 0.05. Red: Higher AD differences, Blue: Lower AD differences, TS-pure: participants with pure Tourette syndrome (TS), OCD: participants with obsessive compulsive disorder, TS+OCD: TS participants with comorbid condition, TS: participants with TS-pure and TS+OCD. [file Image_2.TIFF]

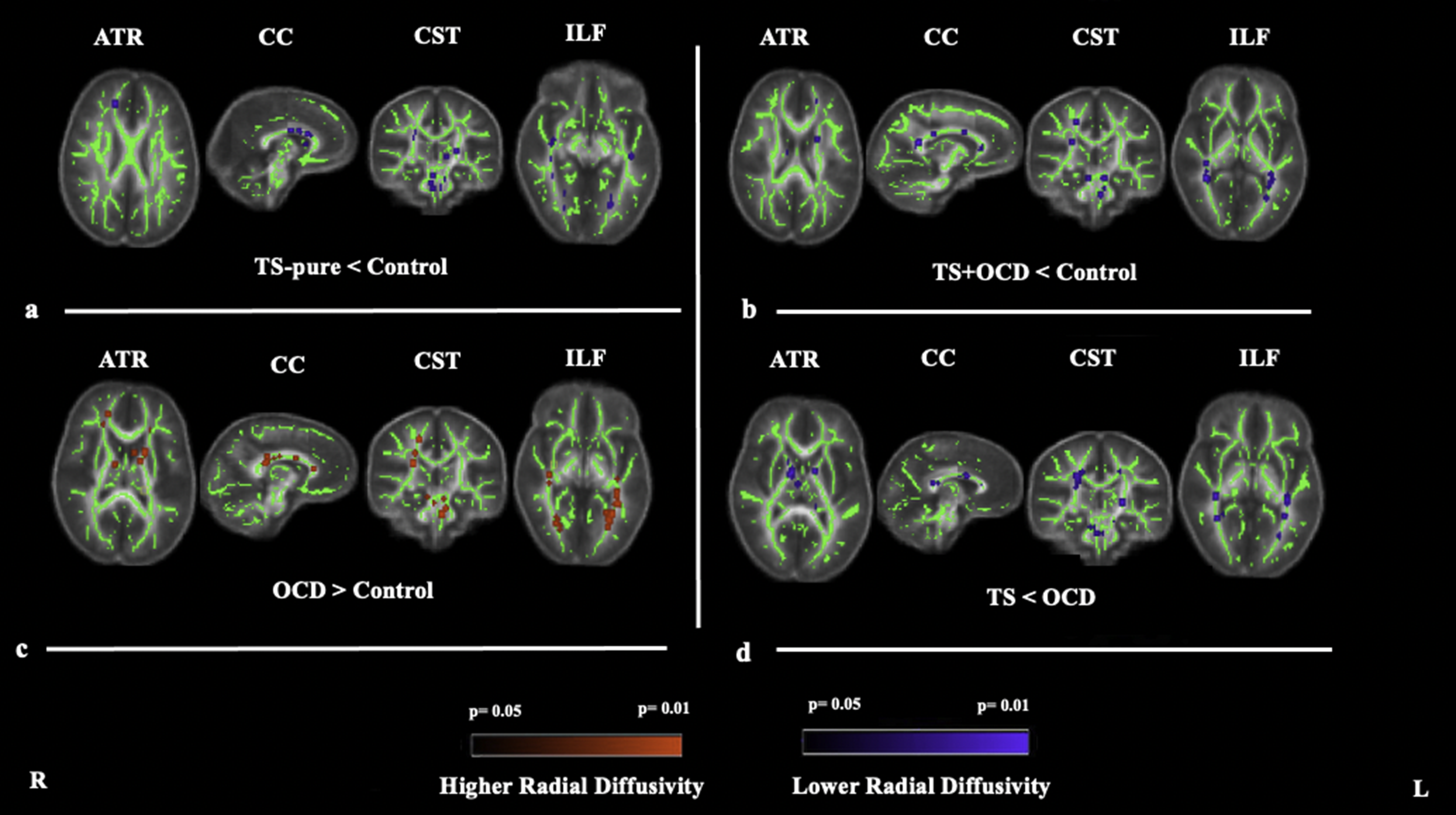

Supplement: Supplementary Figure 3 — Radial diffusivity (RD) differences between (A) TS-pure and controls, (B) TS+OCD and controls, (C) OCD and controls (D) TS and OCD at anterior thalamic radiation (ATR), corpus callosum (CC), corticospinal tract (CST), inferior longitudinal fasciculus (ILF). (A): lower RD in TS-pure than in controls, (B): lower RD in TS+OCD than in controls, (C): higher RD in OCD than in controls, (D): lower RD in TS than in OCD Results were obtained within the mask of ATR, CC, CST, and ILF. Results were presented in the whole brain FA skeleton mask derived from the complete set of participants. RD results were corrected for multiple comparisons at the false discovery rate (FDR) of p < 0.05. Red: Higher RD differences, Blue: Lower RD differences, TS-pure: participants with pure Tourette syndrome (TS), OCD: participants with obsessive compulsive disorder, TS+OCD: TS participants with comorbid condition, TS: participants with TS-pure and TS+OCD. [file Image_3.TIFF]

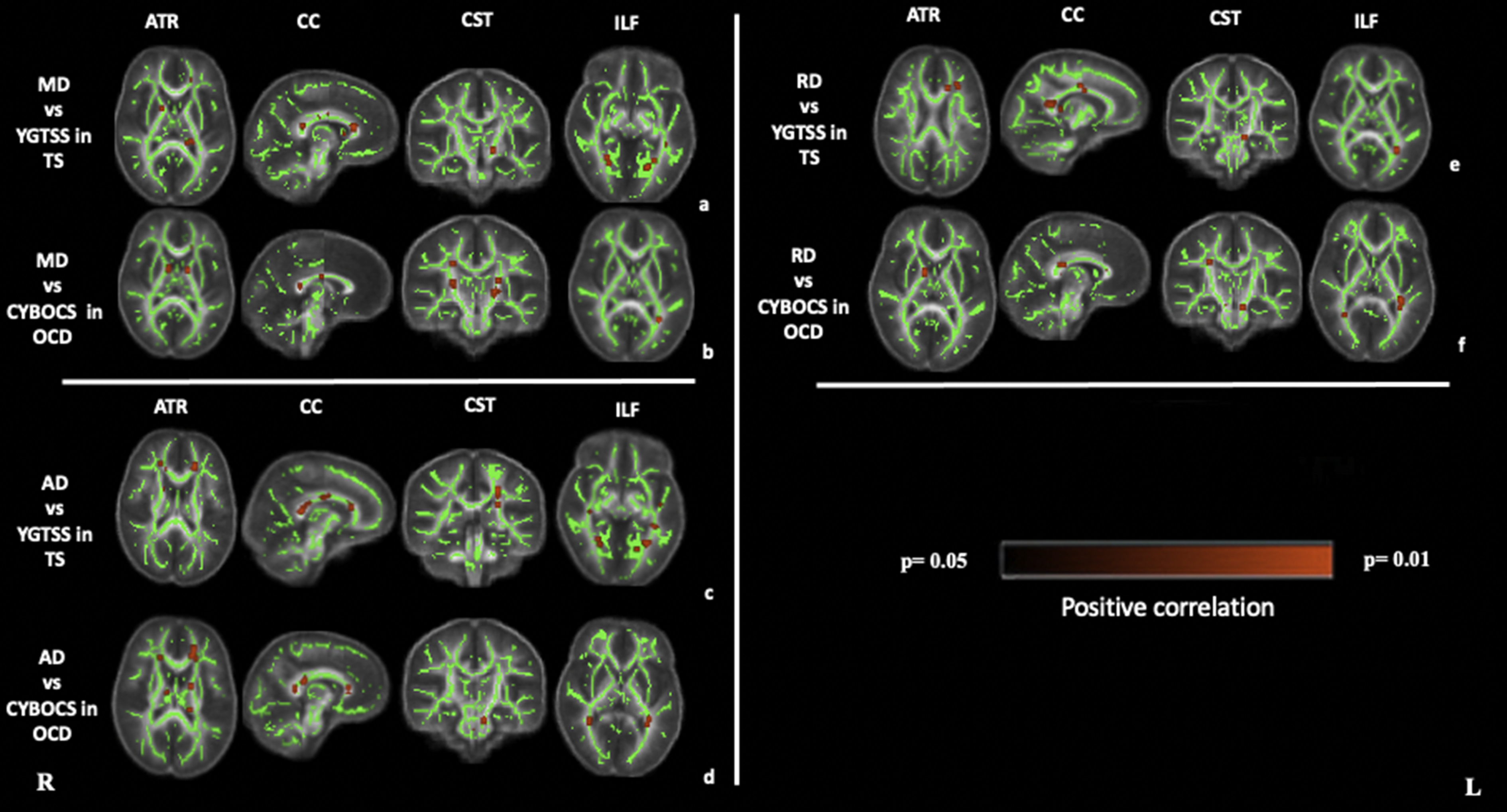

Supplement: Supplementary Figure 4 — Clinical correlations in TS and OCD with YGTSS and CYBOCS at the anterior thalamic radiation (ATR), corpus callosum (CC), corticospinal tract (CST), and inferior longitudinal fasciculus (ILF). (A): positive correlation in TS between MD and YGTSS, (B): positive correlation in OCD between MD and CYBOCS, (C): positive correlation in TS between AD and YGTSS, (D): positive correlation in OCD between AD and CYBOCS, (E): positive correlation in TS between RD and YGTSS, (F): positive correlation in OCD between RD and CYBOCS. Results were presented in the whole brain FA skeleton mask derived from the complete set of participants. Results were corrected for multiple comparisons at the false discovery rate (FDR) of p < 0.05. TS: participants with pure Tourette and comorbid condition [TS+(TS+OCD)]. OCD: participants with obsessive compulsive disorder, YGTSS: Yale Global Tic Severity Scale, CYBOCS: Children's Yale Brown Obsessive-Compulsive Scale. [file Image_4.TIFF]
